# Estimating the Effectiveness of Early Control Measures through School Absenteeism Surveillance in Observed Outbreaks at Rural Schools in Hubei, China

**DOI:** 10.1371/journal.pone.0106856

**Published:** 2014-09-24

**Authors:** Yunzhou Fan, Mei Yang, Hongbo Jiang, Ying Wang, Wenwen Yang, Zhixia Zhang, Weirong Yan, Vinod K. Diwan, Biao Xu, Hengjin Dong, Lars Palm, Li Liu, Shaofa Nie

**Affiliations:** 1 Department of Epidemiology and Biostatistics, School of Public Health, Tongji Medical College, Huazhong University of Science and Technology, Wuhan, China; 2 Department of Health Surveillance and Management, Futian District Center for Disease Control and Prevention of Shenzhen, Guangdong, China; 3 Division of Global Health (IHCAR), Department of Public Health Sciences, Karolinska Institutet, Stockholm, Sweden; 4 School of Public Health, Fudan University, Shanghai, China; 5 Institute of Public Health, Heidelberg University, Heidelberg, Germany; 6 Future Position X (FPX), Gävle, Sweden; University of Hong Kong, Hong Kong

## Abstract

**Background:**

School absenteeism is a common data source in syndromic surveillance, which allows for the detection of outbreaks at an early stage. Previous studies focused on its correlation with other data sources. In this study, we evaluated the effectiveness of control measures based on early warning signals from school absenteeism surveillance in rural Chinese schools.

**Methods:**

A school absenteeism surveillance system was established in all 17 primary schools in 3 adjacent towns in the Chinese region of Hubei. Three outbreaks (varicella, mumps, and influenza-like illness) were detected and controlled successfully from April 1, 2012, to January 15, 2014. An impulse susceptible-exposed-infectious-recovered model was used to fit the epidemics of these three outbreaks. Moreover, it simulated the potential epidemics under interventions resulting from traditional surveillance signals. The effectiveness of the absenteeism-based control measures was evaluated by comparing the simulated datasets.

**Results:**

The school absenteeism system generated 52 signals. Three outbreaks were verified through epidemiological investigation. Compared to traditional surveillance, the school absenteeism system generated simultaneous signals for the varicella outbreak, but 3 days in advance for the mumps outbreak and 2–4 days in advance for the influenza-like illness outbreak. The estimated excess protection rates of control measures based on early signals were 0.0%, 19.0–44.1%, and 29.0–37.0% for the three outbreaks, respectively.

**Conclusions:**

Although not all outbreak control measures can benefit from early signals through school absenteeism surveillance, the effectiveness of early signal-based interventions is obvious. School absenteeism surveillance plays an important role in reducing outbreak spread.

## Introduction

Researchers have recently started paying attention to hysteresis in traditional surveillance systems when detecting infectious disease outbreaks. Scientists have actively explored the possibility of monitoring diseases using pre-diagnosis information since the 1990s. The theory and practice of syndromic surveillance have developed rapidly since the September 11, 2001, attacks on New York and Washington, D.C. Researchers have since found that patients' chief complaints [1], OTC medication sales [2], and school absenteeism [3] can warn of epidemics or infectious disease outbreaks in advance.

Since use of syndromic surveillance systems has increased in developed countries [4], [5], more developing countries and low-resource regions have begun to employ their own versions [6]–[8]. In such areas, traditional disease surveillance faces sizeable challenges: (1) residents live in crowded living environments with poor health conditions, (2) health resources are insufficient to support laboratory diagnoses, and (3) governments cannot afford sufficient education and mass grassroots training to help medical staff recognize new infectious diseases. As a result, residents in these regions are more vulnerable to infectious disease, thereby increasing the urgent necessity for the development of a flexible, convenient, and economically viable syndromic surveillance system for developing countries and low-resource rural regions.

School absenteeism surveillance (SAS) is a common data source for syndromic surveillance. Since schools are gathering places for large groups of young children, they are typical locations for the spread of infectious diseases. School-aged children play an important role in the spread of infectious disease because they are the link between the schools and families [9]. Previous studies on SAS focused on the correlations between it and other surveillance systems [8], [10]–[12]. This paper, however, assesses the role of SAS in three disease outbreaks (varicella, mumps, and an influenza-like illness [ILI]) during the surveillance period.

We fit an infectious dynamic model to these three real epidemics that occurred under interventions resulting from early SAS signals. Moreover, this model was used to simulate corresponding potential epidemics that could occur under the same control measures but from traditional surveillance signals defined by the “National Public Health Emergency Information Reporting and Management Specification (NPHEIRMS)” [13]. Comparing these simulated epidemic datasets generated by the dynamic model, we should be able to estimate the excess preventative effects of the control measures based on the early signals provided by SAS.

## Materials and Methods

### Study area

**Figure 1:**
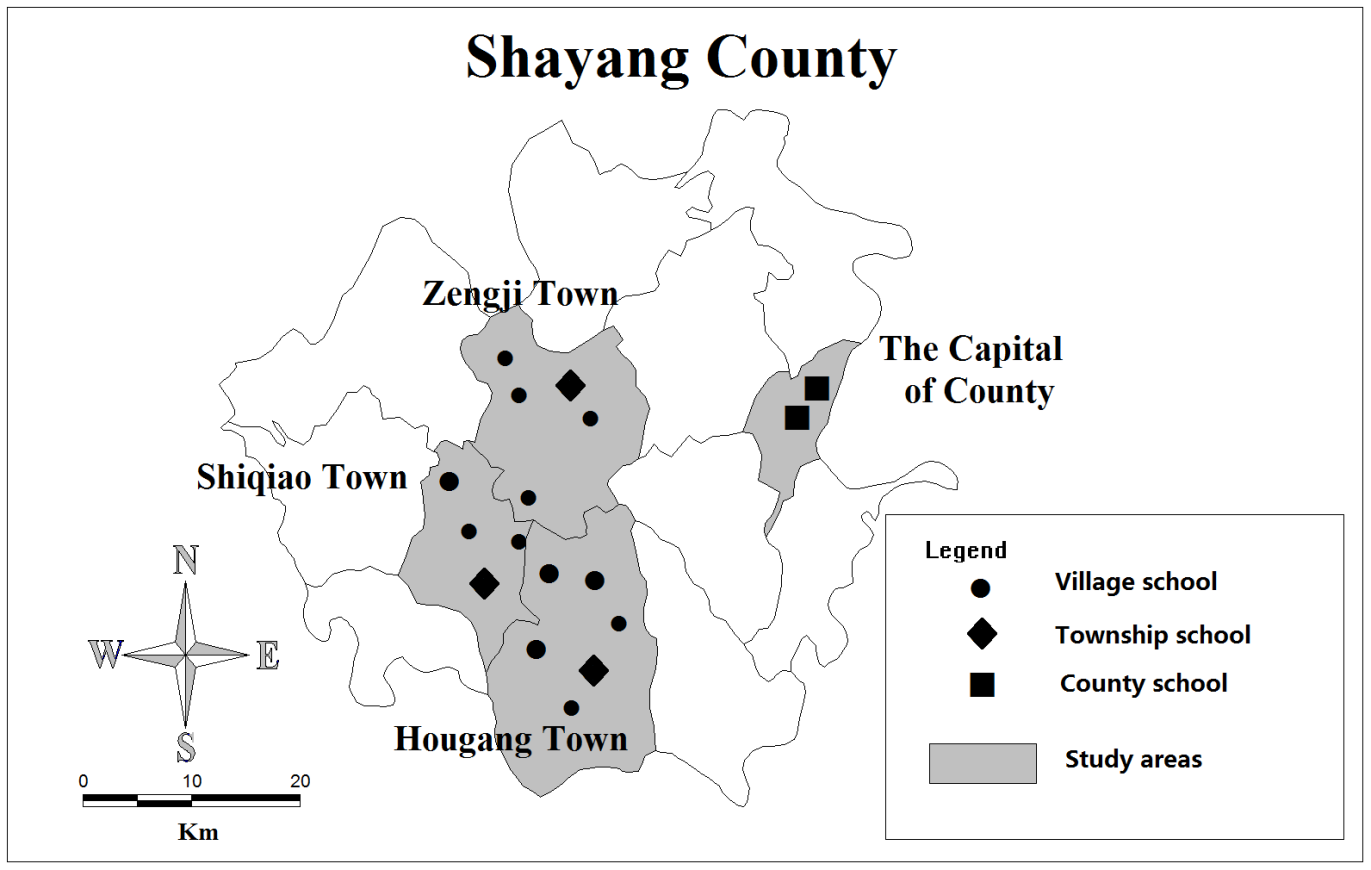
Geographic location of the study areas and monitoring schools.

We conducted the study at 17 primary schools in Shayang County, Hubei, China, from April 1, 2012, to January 15, 2014. We applied a stratified sampling strategy to select the participant primary schools at three levels. All primary schools (2 in total) were selected at the county level in the capital of Shayang. At the township level, a random sampling was applied to obtain 25% of adjacent towns in order to reduce sampling errors as much as possible within the limited funding budget (Zengji, Shiqiao, and Hougang). In the sampled towns, all township-level schools (3 in total) were selected. At the village level, all schools (12 in total) in the sampled towns were selected (see Figure 1). The number of students in each school ranged from 88 to 2,212, for a total of 8,614 students.

### Data collection

Classroom teachers collected absentee information twice daily—in the morning and at noon. If students were absent, the teachers contacted their parents in a timely manner to inquire about the cause of the absence. If the absence was due to illness, the teachers inquired about the specific disease or symptoms. Following this, the teachers summarized all absence information relevant to their classes for a data reporter before 4 PM, and then the reporter submitted the school's absence information to the SAS before 6 PM. These reports included the dates and numbers of absent students, as well as their ages, sexes, classes, addresses, reasons for absenteeism, and diseases or symptoms. In the event that timely communication with the parents was not possible, the reports lacking information concerning the reason for the absence still had to be submitted on the same day. It was possible to contact the parents later and then subsequently supplement the record with the relevant information.

### Quality control

We carried out requisite knowledge and skills training for the schools' teachers. During the study period, administrators conducted daily remote supervision and data checks through the electronic reporting system. Moreover, monthly summaries about data submission quality and feedback were provided to teachers and data reporters to ensure the maintenance of surveillance data quality.

### Response to alarm signals

Once alarm signals emerged, the researchers immediately verified their authenticity using the following steps:

1. Data verification: The researchers checked for errors and duplicate data on the day of the signal, and they assessed whether the students were absent due to the same disease or symptom. In the case of errors, duplicate dates, or different absence reasons, the signal was deemed false and was discarded.
2. Primary judgment: The researchers verified the presence of a spatial correlation among the absent students on the day when the signal occurred. If a correlation was detected, the signal was deemed a suspected signal.
3. Epidemiological investigation: The suspected signal was sent to the local CDC, which then sent investigators to carry out on-site epidemiological investigations aimed at ascertaining the occurrence of an outbreak based on clinical diagnosis and the contact histories of the absent students. Once the outbreak was verified, corresponding measures were immediately taken to control disease spread.

### Intervention

We formulated a standard operation procedure (SOP) of comprehensive intervention against verified outbreaks as follows:

1. Isolation: The patients were required to be quarantined either at home or in the hospital to reduce contact with others. The patients returned to school after receiving a hospital-issued post-recovery clean health certificate.
2. Disinfection: The classrooms and daily supplies were disinfected at least twice a day by wiping or spraying a solution containing 500 mg/L of chlorine with an action time of 30 minutes.
3. Ventilation: This occurred for at least 2 hours every day to keep the air fresh in all classrooms.
4. Health inspection: The teachers inspected all students for relevant symptoms in the morning and at noon. The teachers reported the manifestation of any suspected cases and immediately isolated the student for observation.
5. Provision of liquid soap: The schools provided liquid soap at the sinks on every floor to encourage students to wash their hands and to pay attention to personal hygiene.
6. Health education: We publicized disease prevention knowledge by means of broadcasts and blackboard newspapers to educate all students.

All these pre-specified control measures were taken for all three disease outbreak events in our study.

### Alarm signals

#### School absenteeism surveillance

We set up an EARS∼3Cs model as the early warning algorithm in the SAS system. This is popular among a wide variety of health departments, and it is intended for use as a cumulative sum-like method consisting of three algorithms, C1, C2, and C3, which show increasing levels of sensitivity (C3 being the most sensitive). The C1 baseline is obtained from the previous 7 days in closest proximity to the current day (Day t-7 through Day t-1). C2 uses a 7-day baseline on Day t-9 through Day t-3. C3 is the sum of the C2 values for the past 3 days. The EARS∼3C statistical values are relative numbers that reflect the fluctuation volatility of time-series data, which have taken the baseline population size into consideration. This statistic can be written as follows:
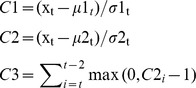
where x_t_ is the count of cases at Day t and *µ_t_* and *σ_t_* are the moving sample mean and standard deviation at baseline, respectively. The EARS methodological details are described elsewhere [14].

According to the U.S. Centers for Disease Control and Prevention's (CDC) experience with the EARS system, the threshold values of C1, C2, and C3 in this model should be set as 3, 3, and 2, respectively [14]. C1, C2, and C3 are set to automatically and simultaneously analyze the daily data for each primary school and mark the data that exceed threshold. Additionally, in order to judge the spatial associations among absentees, we set a minimum requirement of 3 absentees for an alarm signal to occur. Thus, a combined threshold was used to generate the alarm signals. If the number of school absences exceeded 3 on any given day and the marks occurred in two or more algorithm models on the same day (C1C2, C1C3, C2C3, or C1C2C3), the system sent out an alarm signal.

#### Traditional surveillance

China's Ministry of Health formulated the National Public Health Emergency Information Reporting and Management Specification (NPHEIRMS) [13] in 2006. This specification defined the reporting standards for infectious disease outbreaks. All legal entities (including schools) are obligated to monitor diseases and to report possible signals in a timely manner. Specifically, when the number of disease cases in one school exceeds the weekly reporting standard, the school has to report to the local health administrative department within two hours. Then the department carries out an on-site investigation and takes relevant outbreak control measures. According to the reporting standards of NPHEIRMS, the thresholds for the three diseases discussed in this paper are as follows: 10 varicella cases/week, 10 mumps cases/week, and 30 influenza-like illness (ILI) cases/week.

### Estimation of early control measure effectiveness

The theoretical method for estimating the effectiveness of early control measures entails comparing the infection attack rate under the earlier intervention versus traditional intervention (the control group). Once early intervention is implemented, however, it is impossible to obtain the data through traditional intervention in the observed events. In order to estimate the effectiveness of early interventions, a dynamic model of infectious diseases was introduced to simulate the control group (the potential epidemic under traditional interventions) [15]. Thus, the effectiveness of early control measures can be estimated by comparing simulated epidemic datasets (generated by the dynamic model) between early and traditional interventions. The extra protective rate (EPR) can be used to evaluate the effectiveness of the early control measures. The equation for this rate is as follows:
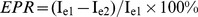
where I_e1_ is the estimated attack rate under the control measures based on traditional surveillance (NPHEIRMS), and I_e2_ is the attack rate under the earlier control measures based on the SAS. Both I_e1_ and I_e2_ are calculated using fitted epidemic datasets through the dynamic model.

### Impulse susceptible-exposed-infectious-recovered model

The susceptible-exposed-infectious-recovered (SEIR) model is a basic epidemic dynamic model [15]. The basic SEIR model imitates the four main health states of disease progression. Susceptible individuals (S) become exposed (E) at rate *β* due to contact with infected individuals. Then those exposed become infected individuals (I) at rate *ω*. Finally, infected individuals enter the recovery state (R) at rate *γ* [16], [17]. The following equations model this process:
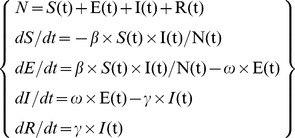
where *N* is the total number of individuals. S(t), E(t), I(t), and R(t) are the numbers of individuals at time *t* in each of the four states. The ratios of 1/*ω* and 1*/γ* are the mean incubation and infectious periods, respectively. *β* is the transmission rate, which reflects a disease's diffusion intensity. This is usually measured by the reproductive number (*R_0_*), which refers to the number of secondary cases for each primary case:


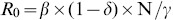
, where *δ* is the population's positive antibody rate.

Unlike general surveillance systems, SAS provides discontinuous information because weekends, holidays, and vacation periods (e.g., winter and summer) fracture surveillance continuity. School class suspension largely reduces the chances for mutual student contact, and plays a notable role in hindering the transmission and spread of infectious diseases. Therefore, to improve the schools' regular class suspensions, we added a time control variable for *β* into the basic SEIR model to construct an impulse SEIR model. The time control variable for *β* can be defined thus:
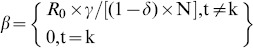
where *k* represents the dates schools were closed, such as weekends, holidays, or vacations.

### Parameter

To fit the impulse SEIR model to the real epidemics of these three outbreaks in schools under interventions resulting from SAS signals, we defined the parameter values of *R_0 before intervention_*, *R_0 after intervention_*, *ω*, *γ*, and *δ* for varicella, mumps, and ILI.

We obtained the possible ranges of *R_0 before intervention_* for the three diseases from those established in the existing literatures, such as 7.0–12.0 for varicella[18], 3.8–18.2 for mumps[19], and 1.6–3.0 for seasonal influenza[20].

The *R_0 after intervention_* could be estimated according to the realistic epidemic curves in each outbreak event [18]:
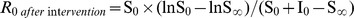
where S_0_ and S_∞_ are the number of susceptible individuals at the start and the end of the outbreak, respectively; I_0_ represents the number of cases at the beginning.

We also estimated the parameters of *ω* and *γ*, according to the average incubation and infectious periods of each disease sourced from relevant literature [21]–[23] (Table 1). For the study areas that were rural and resource-poor, the local CDC often lacked the ability to perform serologic tests for positive antibody rate *δ* among the population. Thus, we searched for relevant published data [24]–[30] to estimate the possible ranges of *δ* for the most common virus subtypes among the Chinese school-aged population from 2011 to 2013.

**Table 1:** Parameters used in the simulation.

Parameter	varicella	Mumps	influenza
*R_0 before intervention_*	7.0–12.0	3.8–18.2	1.6–3.0
*R_0 after intervention_*	1.0	0.9	0.7
*ω*	0.08	0.09	0.5
*γ*	0.15	0.07	0.25
*δ*	0.42–0.69	0.55–0.87	0.14–0.69

*δ* is the possible range of positive rate of antibody in Chinese school-aged children during 2011∼2013, derived from the literature.

### Ethics Statement

Written informed consent was obtained from the participants, including the children's parents. All participants and patients were de-identified, and only aggregated data was analyzed. The personal identification information did not appear in the final database. This study has been ethically approved by the Institutional Review Board of Tongji Medical College.

## Results

The SAS ran for 655 days (April 1, 2012, to January 15, 2014). During this period, there were 337 (51.5%) monitoring days and 318 (48.5%) non-monitoring days (i.e., schools were closed for weekends, holidays, and vacations). A total of 1,702 (1,356 due to illness) reports were recorded, and an average of 5.1 (4.0 due to illness) students were absent from school each monitoring day. The proportion of missing data due to failure to contact parents was acceptable, accounting for only 4.8% (81/1702) of the total collected data.

**Figure 2:**
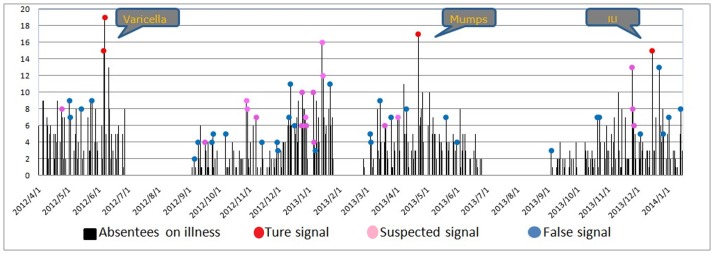
The three types of signals in all schools generated by school absenteeism surveillance system. Signals were generated by the EARS∼3Cs.

**Figure 3:**
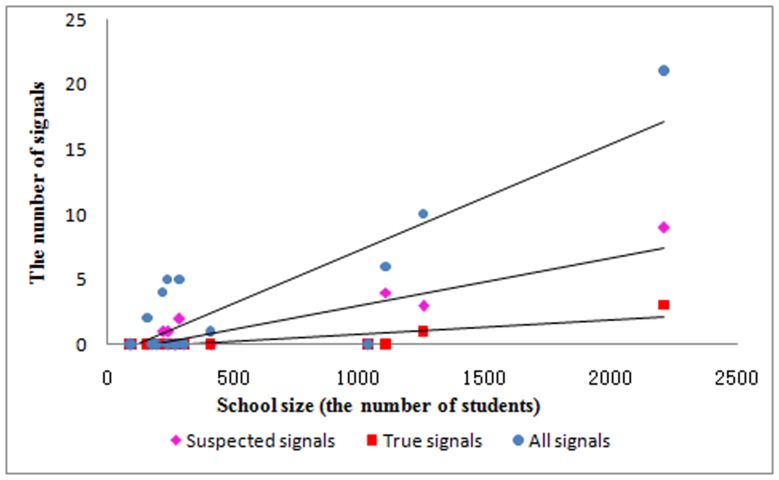
The correlation between school size (the number of students) and the number of signals.

By using the EARS∼3Cs algorithm for absenteeism data due to illness in each school, the system detected 52 signals in 8 schools. Among these, 32 signals were excluded due to either false/duplicate records or insufficient spatial association. The staff from the local CDC carried out epidemiologic investigations for the 20 remaining suspected signals. Consequently, 4 signals (2 varicella, 1 mumps, and 1ILI; see Figure 2 and Table 2) generated from 3 outbreak events were verified according to clinical diagnosis and contact history. In addition, Figure 3 illustrates that school size was positively correlated with the number of signals (r = 0.86 for all signals, r = 0.89 for suspected signals, r = 0.85 for true signals with all *P*<0.001).

**Table 2:** Summary of absentees, signals, and outbreaks in each monitored school.

School	N of students	N of absentees	Average absentees per day	N of absentees for illness	Average absentees for illness per day	N of signals	N of suspected signals	N of true signals	Outbreaks	Diseases
1	88	10	0.03	7	0.02	0	0	0	0	
2	99	27	0.08	17	0.05	0	0	0	0	
3	163	31	0.09	14	0.04	2	0	0	0	
4	182	35	0.10	22	0.07	0	0	0	0	
5	201	7	0.02	6	0.02	0	0	0	0	
6	223	129	0.38	86	0.26	4	1	0	0	
7	242	1	0.01	1	0.00	0	0	0	0	
8	243	55	0.16	51	0.15	5	1	0	0	
9	267	19	0.06	13	0.04	0	0	0	0	
10	277	37	0.11	20	0.06	0	0	0	0	
11	290	134	0.40	114	0.34	5	2	0	0	
12	309	5	0.01	4	0.01	0	0	0	0	
13	414	56	0.17	44	0.13	1	0	0	0	
14	1039	31	0.09	23	0.07	0	0	0	0	
15	1108	49	0.15	48	0.14	6	4	0	0	
16	1257	124	0.37	118	0.35	10	3	1	1	ILI
17	2212	952	2.82	768	2.28	21	9	3	2	varicella, mumps
Total	8614	1702	5.05	1356	4.02	52	20	4	3	

There were 337 monitoring days during the whole period. Signals were generated by the EARS∼3Cs algorithm. Suspected signals were verified by data checking and spatial judgment. True signals were verified by epidemiologic investigation and clinical diagnosis.

**Figure 4:**
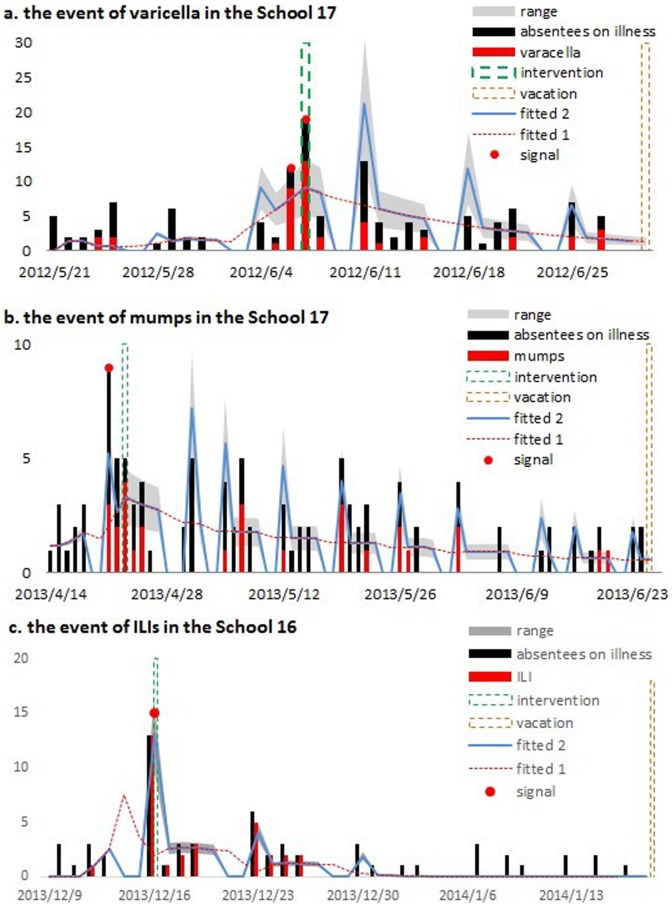
Model fit of epidemics in (a) varicella outbreak, (b) mumps outbreak, and (c) influenza-like illness outbreak. Vertical dash lines indicate the timing of control measures and vacations. Red dots indicate alarms by warning algorithm. ‘Fitted 1’ is the fitted line generated directly using impulse SEIR model; ‘Fitted 2’ is the fitted line adjusted by the effect of suspending reporting on weekends.

The varicella outbreak in School 17 (Figure 4a) had 41 reported cases (mean age  =  8.4 years old, 18 boys) distributed among 7 classes. More than 50 percent (22) of the cases were concentrated in a single class. Signals were generated continually on June 6 and 7, 2012. The first case could be traced back to May 24, 2012. The pre-specified control measures were carried out on June 8 and lasted for 3 days.

The mumps outbreak in School 17 (Figure 4b) consisted of 28 reported cases (mean age  = 9.1 years old, 15 boys) in 10 classes, with a significant proportion of (7) cases originating from a single class. The first case could be traced back to April 22, 2013. The alarm signal was noted on the same day. The pre-specified control measures were performed on April 24, 2013 and lasted for 3 days.

For the ILI outbreak in School 16 (Figure 4c), 31 reported cases (mean age  = 6.9 years old, 21 boys) occurred in 8 classes, and a significant proportion of cases (13) came from a single class. The first case could be traced back to December 12, 2013, and the signal was generated on December 16, 2013. Due to the number of cases (13) that arose suddenly on this day, school administrators responded immediately, began the pre-specified control measures on the next day, and kept them going for the next 4 days.

We next fit the impulse SEIR model to the data that resulted from the school absenteeism surveillance (Figure 4). The dashes labeled “Fitted 1” are the fitted lines that were generated directly through the impulse SEIR model. Actually, SAS is unable to record the absent students on weekends due to school closure; thus the infected students who developed relevant symptoms on the weekends were recorded on the following Monday. Taking this situation into consideration, we adjusted “Fitted 1” by replacing the number of cases on weekends with “0” and adding these cases occurring on weekends with those of the following Monday (“Fitted 2”). We found that the impulse SEIR model fit the real epidemics to a large extent (Figure 4), although some systematic errors were likely to exist between the reported and the fitted data (e.g., students with mild symptoms may not be absent from school, particularly before the signals were generated; and biased absence reasons may be obtained in the process of enquiry for students' parents).

**Figure 5:**
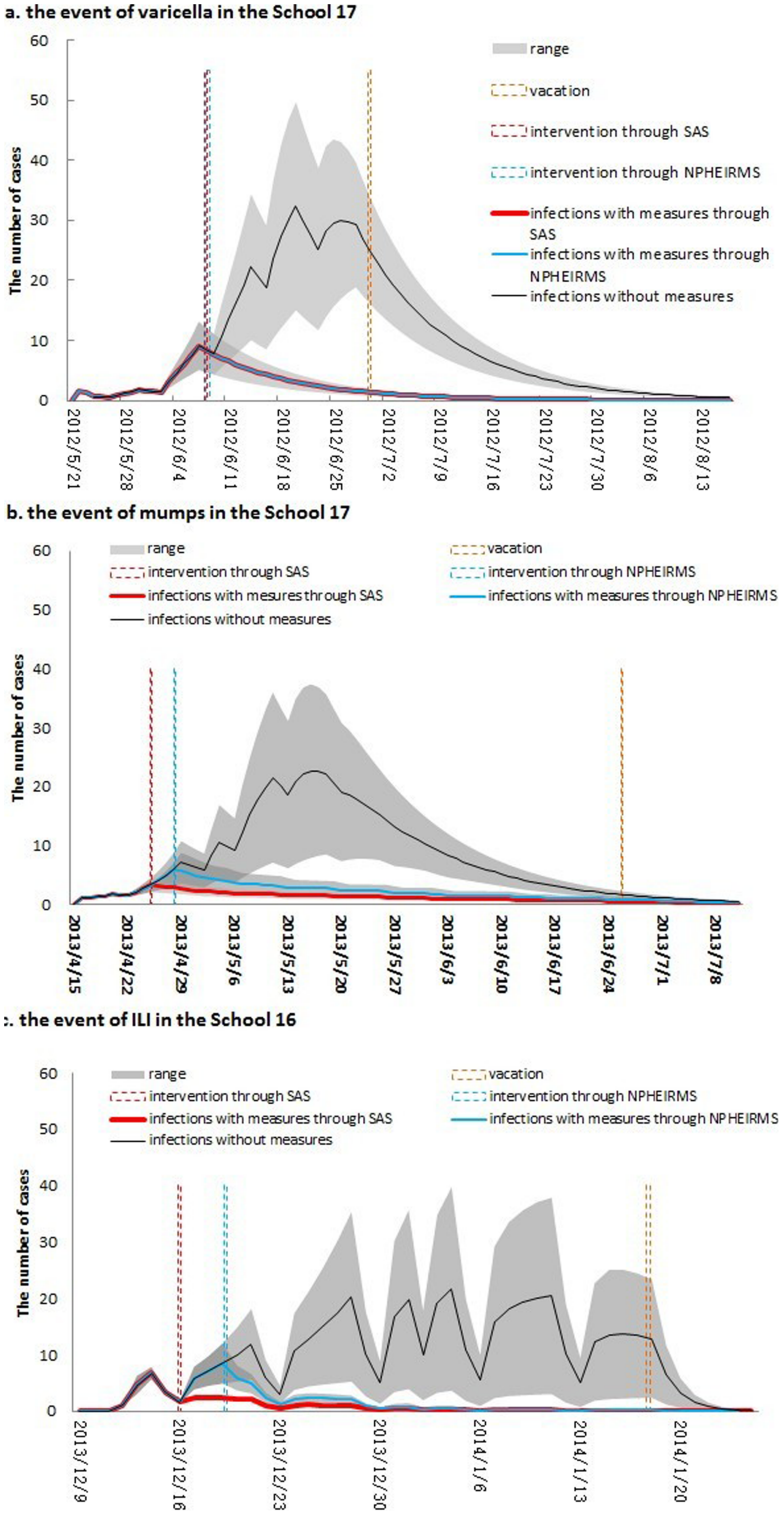
The simulated effects of control measures using impulse SEIR model in three outbreaks in schools. Although the surveillance for absenteeism was stopped after vacation, we still estimated the simulated infections until the epidemic faded away. SAS: school absenteeism surveillance; NPHEIRMS: National Public Health Emergency Information Reporting and Management Specification.

**Figure 6:**
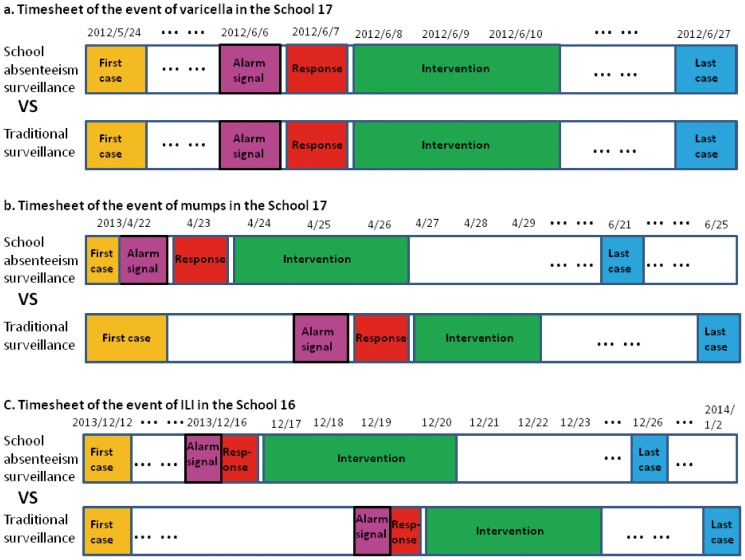
The comparison between the timesheets of outbreak events through school absenteeism surveillance and traditional surveillance. Time points through school absenteeism surveillance were recorded according to the actual observations, and time points through traditional surveillance were recorded according to the simulated epidemics by impulse SEIR model.

We simulated the potential disease epidemics without intervention (Figure 5, black lines) and with intervention based on the traditional NPHEIRMS (Figure 5, blue lines). For the varicella outbreak, the NPHEIRMS generated the signal at the same time the SAS did (the vertical dashed lines indicating the intervention timing of the SAS and the NPHEIRMS overlapped). However, for the outbreaks of mumps and ILI, the NPHEIRMS generated the signals 3 days, in average, after the SAS did. Figure 6 shows the comparisons between the timing of outbreak events based on SAS and traditional surveillance.

Using the fitted datasets of these three outbreaks under the interventions resulting from the SAS signals, we estimated the attack rates of varicella, mumps and ILI outbreaks as 7.1%–28.3%, 5.0%–41.8%, and 4.6%–14.6%, respectively. With the same alarm response time and intervention strength, the attack rates of these three outbreaks under traditional NPHEIRMS control measures would reach 7.1%–28.3%, 6.2%–62.9%, and 6.7%–22.3%, respectively (Table 3). Although the SAS failed to show obvious advantages (excess protection rate of 0.0%) compared with the NPHEIRMS for the varicella outbreak, it sent out earlier alarm signals in the mumps and ILI outbreaks. The excess protection rates indicated that the advanced interventions resulting from early signals could reduce the potential mumps and ILI cases by 15.3%–44.1% and 29.0%–37.0%, respectively.

**Table 3:** Estimation of control measures' effectiveness through SEIR models for SAS and NPHEIRMS in three school outbreaks.

Event	R_0 before intervention_	*δ*	N. of susceptible	SAS			NPHEIRMS			Time on SAS ahead of NPHEIRMS (day)	EPR of early measures through SAS (%)
				Signal	N. of infections	Attack rate (%)	Signal	N. of infections	Attack rate (%)		
Varicella	7.0	0.42	1283	2012/6/6	91	7.1	2012/6/6	91	7.1	0	0.0
		0.69	686	2012/6/6	88	12.8	2012/6/6	88	12.8	0	0.0
	12.0	0.42	1283	2012/6/6	209	16.3	2012/6/6	209	16.3	0	0.0
		0.69	686	2012/6/6	194	28.3	2012/6/6	194	28.3	0	0.0
Mumps	3.8	0.55	1017	2013/4/22	51	5.0	2013/4/25	63	6.2	3	19.0
		0.87	294	2013/4/22	50	17.0	2013/4/25	59	20.1	3	15.3
	18.2	0.55	1017	2013/4/22	147	14.5	2013/4/25	263	25.9	3	44.1
		0.87	294	2013/4/22	123	41.8	2013/4/25	185	62.9	3	33.5
ILI	1.6	0.14	1081	2013/12/16	50	4.6	2013/12/20	72	6.7	4	30.6
		0.69	390	2013/12/16	49	12.6	2013/12/20	69	17.7	4	29.0
	3.0	0.14	1081	2013/12/16	58	5.4	2013/12/18	92	8.5	2	37.0
		0.69	390	2013/12/16	57	14.6	2013/12/18	87	22.3	2	34.5

SAS represents school absenteeism surveillance. NPHEIRMS is the National Public Health Emergency Information Reporting and Management Specification. EPR is the extra protective rate.

## Discussion

We analyzed three observed disease outbreaks in primary schools and compared the results with numerical simulations derived from the impulse SEIR model. Although, the SAS showed no obvious advantages in the varicella outbreak, it sent out alarms an average of three days ahead of the NPHEIRMS for the mumps and ILI outbreaks, which were effectively controlled by the early investigations and interventions of the local CDC.

In fact, the SAS's ability to send out a warning signal ahead of the NPHEIRMS may have been linked to the early intensity of the outbreak. The current NPHEIRMS threshold values are fixed, with no fluctuations of monitoring data taken into account, and are defined according to the number of cases occurring within one week (e.g., 10 varicella cases/week, 10 mumps cases/week, and 30 ILI cases/week). If the number of cases increases rapidly during the early stage of the epidemic, the number of cumulative cases could easily exceed the NPHEIRMS thresholds, allowing the NPHEIRMS to also send timely warning signals. However, if the number of cases increases gradually during the early stage of the epidemic, the NPHEIRMS may send a warning only when the accumulated weekly cases exceed the threshold. In this case, SAS can send out early alarm signals according to the monitored data fluctuations. Therefore, it is much more sensitive to disease outbreaks and sends out earlier warning signals much more easily.

Time is key in controlling the spread of outbreaks. An early warning signal offers the opportunity for early intervention, but whether a successful early intervention can be conducted depends on the signal's accuracy. An early outbreak warning may be realized through syndromic surveillance based on disease-related non-specific data; but the biggest disadvantage to this is that a large number of false signals were generated. It is, therefore, essential to screen the signals sent out from any particular syndromic surveillance system. Signals can be verified by checking data quality and the spatial relationships between cases, and then undertaking epidemiological investigations.

Among the three disease outbreaks in our study, the response times differed. It is noteworthy that the measures in the ILI outbreak were taken by the school administrators themselves rather than the local CDC staff. The 13 students with influenza-like symptoms were absent on the day of the warning signal; and 10 of these were from the same class. The school administrators attached great importance to this type of localized mass absence and took control measures themselves on the next day. School administrators with stronger syndromic surveillance awareness could speed up the response time to signals, allowing necessary additional time for early interventions. Therefore, it is helpful to carry out SAS and disease control education for both teachers and school administrators.

The key to evaluating measure effectiveness lies in how the appropriate control group is chosen. In the actual outbreak, once an intervention is performed, the surveillance system can no longer monitor the potential cases without intervention. Moreover, it is almost impossible to find another similar outbreak event to use for the control group. Therefore, the simulation of a control group carried out through an epidemic dynamic model is one of the best ways to evaluate measure effectiveness. Given the impact of weekends and holidays on school disease transmission, situations with an intervention based on traditional NPHEIRMS are simulated through the impulse SEIR model, which is in line with the characteristics of discontinuous surveillance of SAS and automatically turns the parameter of *β* to 0 while schools are closed, thus more realistically reflecting the school's disease transmission.

Infectious disease epidemics are closely related to population density. During the SAS surveillance period, the signals mainly occurred in large schools. The three disease outbreaks came from the two largest schools (Schools 16 and 17). Schools are the main places for social activities among school-aged children, and the increased contact among students in a comparatively closed environment greatly contributes to the spread of infectious diseases. Therefore, larger schools are more vulnerable to disease outbreaks due to more transmission agents and more frequent close contact. This suggests that SAS is more necessary in larger schools to detect possible disease outbreaks at the early stage.

There are still some problems with carrying out SAS in low-resource rural areas. First, laboratory tests for disease confirmation are not usually available in these areas. Cases can, therefore, only be confirmed through clinical diagnosis at an outbreak's earliest stages. On the other hand, the uncertainty of virus subtypes also influences the accuracy of numerical simulation results. Second, accurate data for the seropositive rate of antibodies was not obtained, nor were sufficient funds available to test all students. Therefore, we could only use the possible ranges for seropositive antibody rates from the existing literature in order to estimate the number of susceptible individuals. Finally, although the researcher remotely supervised each school's report quality, there were still some missing reports resulting in the systematic errors between the realistic and fitted data. Therefore, increasing local teachers' awareness of syndromic surveillance in the future is also important for school absenteeism surveillance.

Although the SAS faced some challenges in the low-resource rural areas, it was able to send out early warning signals. The early control measures based on the SAS played an important role in preventing and controlling the infectious diseases, thus, the SAS can be feasibly and effectively used in rural China.

## Supporting Information

Table S1 **The original school absenteeism surveillance data from all 17 primary schools in the study areas.**
(XLS)
